# Analysis of knowledge, attitudes and practices regarding exercise among type 2 diabetes patients and influencing factors: a cross-sectional study

**DOI:** 10.3389/fmed.2026.1766153

**Published:** 2026-02-10

**Authors:** Detian Liu, Hongzhen Xie, Huang Chen, Mengwei Jiang, Duo Liu, Ting Shu, Zijun Yuan, Xiaoqi Fan, Jianjing Wang

**Affiliations:** 1 School of Nursing, Guangdong Pharmaceutical University, Guangzhou, China; 2 Department of Health Medicine, General Hospital of Southern Theater Command of PLA, Guangzhou, China; 3 Department of Endocrinology, General Hospital of Southern Theater Command of PLA, Guangzhou, China; 4 Xianning Vocational Technical College, Xianning, China; 5 School of Nursing, Southern Medical University, Guangzhou, China

**Keywords:** exercise therapy, health behavior, influencing factors, Knowledge-Attitude-Practice, type 2 diabetes

## Abstract

**Objective:**

This study investigates exercise-related knowledge, attitude, and practice (KAP) status and its determinants among type T2DM, with the goal of offering practical references to optimize the implementation of exercise-based interventions for this patient group.

**Methods:**

A questionnaire survey was conducted using cluster sampling among 383 hospitalized patients with type 2 diabetes in the endocrinology departments of seven tertiary hospitals across Guangdong, Guangxi and Hubei provinces, China. The study analyzed the current status and influencing factors of patients’ knowledge, attitudes and practices regarding physical activity.

**Results:**

Among the T2DM participants, the overall exercise KAP score reached 122.89 ± 24.33, corresponding to a score rate of 66.43%. Specifically, the exercise knowledge subscale obtained 49.10 ± 13.82 points (score rate: 54.56%), the exercise attitude subscale yielded 26.44 ± 7.66 points (score rate: 66.1%), and the exercise practice subscale achieved 47.34 ± 12.55 points (score rate: 67.63%). Multivariate linear regression analysis revealed that long-term residence, educational attainment, monthly income, complications, regular exercise habits, diabetes exercise health education, and family support are related factors of exercise Knowledge-Attitude-Practice levels in T2DM patients (*R*^2^ = 0.284, *P*<0.05).

**Conclusion:**

Exercise KAP levels among T2DM patients are generally suboptimal, necessitating timely targeted interventions. The key populations for exercise-focused health education include those with lower educational background, limited income, rural household registration, and no diabetes-associated complications. Tailoring exercise health education to the assessment findings, boosting family-based supportive measures, and encouraging peer communication among patients can effectively elevate their exercise-related KAP levels.

## 1 Introduction

Type 2 diabetes mellitus (hereafter referred to as T2DM) has become one of the most rapidly growing public health concerns (1), with China’s diabetic population exceeding 140 million, ranking second globally (2). As one of the five pillars of diabetes management, exercise therapy holds a significant position in the comprehensive care of T2DM patients due to its high efficacy, low cost, and minimal adverse effects (3). However, the current exercise status of T2DM patients both domestically and internationally remains concerning. Surveys indicate that few individuals with diabetes maintain regular exercise habits, with participation rates below 10%. Only 25% of adult T2DM patients meet the minimum physical activity standards recommended by the World Health Organization (4, 5). Exercise intervention therapy remains a focal point in diabetes prevention and management (6), with nurses serving as indispensable members of exercise management teams for T2DM populations. In recent years, nursing professionals have explored exercise outcomes, exercise programmes, exercise-related fears, and the application of internet technologies across diverse T2DM subgroups. Nevertheless, overall, research by nurses on exercise for T2DM patients remains limited (7–9). Studies on patients’ knowledge, attitudes, and practices (KAP) have predominantly focused on diabetes disease management (10) and dietary surveys (11), with a notable absence of research on exercise-specific KAP. Assessing current status is a prerequisite for personalized exercise interventions and constitutes a vital component of diabetes nursing management. Previously, addressing the absence of a dedicated instrument for assessing exercise-related knowledge, attitude, and practices among T2DM patients in China, our research group developed the “T2DM Patient Exercise Knowledge, attitude, and Practices Scale” (12). This study employs this scale as its research tool to investigate the current status of exercise-related knowledge, attitude, and practices among T2DM patients and to preliminarily identify the factors associated with these aspects. This provides a reference basis for nurses in developing personalized exercise health education programmes.

## 2 Subjects and methods

### 2.1 Study population and sample size calculation

Between April and July 2025, a multicenter cross-sectional study was conducted in seven tertiary hospitals with established endocrinology departments located in Guangdong, Guangxi, and Hubei provinces, China. A cluster sampling strategy was adopted, with each hospital serving as a primary sampling unit. Within each participating hospital, hospitalized patients with type 2 diabetes mellitus (T2DM) admitted to the endocrinology department during the study period were consecutively recruited. Sample size estimation followed established recommendations for questionnaire-based studies, requiring 5–10 participants per questionnaire item, with an additional 10% allowance for potential nonresponse. Given that the exercise Knowledge-Attitude-Practice (KAP) questionnaire comprised 21 items, the minimum required sample size ranged from 116 to 231 participants. Ultimately, a total of 383 eligible patients were enrolled, exceeding the minimum requirement and providing adequate sample size for subsequent multivariable analyses.

### 2.2 Inclusion and exclusion criteria

Participants were eligible for inclusion if they met the following criteria: (1) a confirmed diagnosis of T2DM according to the American Diabetes Association (ADA) criteria (13); (2) no diagnosed mental disorders and sufficient hearing and verbal communication ability to complete the questionnaire; and (3) voluntary participation with provision of written informed consent. Patients were excluded if they (1) had medical contraindications to exercise or (2) were unable or unwilling to cooperate with the study procedures.

### 2.3 Research tools

#### 2.3.1 General information questionnaire

Based on the research objectives and a review of the literature, a general information questionnaire was designed in conjunction with clinical practice. It included: ① Socio-demographic characteristics: age, gender, marital status, educational attainment, occupation, long-term residence, housing status, income level. ② Lifestyle: sedentary habits, exercise routines. ③ Disease characteristics: body mass index, duration of illness, presence of comorbidities, family history, number of hospitalisations. ④ Exercise management characteristics: participation in diabetes exercise health education programmes; management or follow-up of exercise regimen by healthcare professionals; development of personalized exercise plans by healthcare professionals; presence of family or friends providing exercise encouragement.

#### 2.3.2 Exercise assessment scale for T2DM patients

Developed by Liu Duo (6), this 37-item scale comprises three dimensions: exercise knowledge (15 items), exercise beliefs (8 items), and exercise behavior (14 items). All items are single-choice questions using a 5-point Likert scale (5 = Strongly Agree, 4 = Agree, 3 = Neutral, 2 = Disagree, 1 = Strongly Disagree) with accompanying instructions. This self-report questionnaire requires T2DM patients to select the option most accurately reflecting their personal circumstances. The total score ranges from 37 to 185 points, with higher scores indicating better exercise related performance. Total scores of 37–124 indicate poor physical activity, 125–144 indicate fair, 145–159 indicate good, and 160–185 indicate excellent. The scale’s Cronbach’s *α* coefficient is 0.966; in this study, the Cronbach’s α coefficient was 0.931. To enhance transparency and reproducibility, representative items from each dimension of the Type 2 Diabetes Mellitus Exercise Knowledge-Attitude-Practice (KAP) Scale are provided in Supplementary Table S1. The full scale, including item wording, response options, and scoring instructions, is available as Supplementary material upon reasonable request from the corresponding author.

### 2.4 Data collection and quality control

Data collection was conducted using Wenjuanxing, employing standardized instructions with one-to-one guidance during completion. Respondents accessed the questionnaire by scanning a QR code. All questions were designated as mandatory to ensure completeness; submission was only permitted after all questions were answered, with backend settings restricting each account to a single submission. Prior to the survey, all investigators underwent standardized training to familiarize themselves with the questionnaire content and survey procedures. During data collection, the purpose of the survey and completion requirements were explained in detail to patients, who were encouraged to provide truthful responses. Following questionnaire collection, responses exhibiting obvious logical inconsistencies were screened and supplemented through follow-up interviews to ensure data quality.

Prior to fitting the multivariable linear regression model, key model assumptions were evaluated. Linearity between each independent variable and the dependent variable (total exercise KAP score) was assessed using scatterplots and partial regression plots, which indicated no substantial departures from linear relationships.

The distribution of residuals was examined using histogram and normal P–P plots. Although minor deviations from perfect normality were observed, particularly in the distribution tails, the residuals were approximately symmetrically distributed with a mean close to zero. Given the relatively large sample size (*n* = 383), these minor deviations were considered acceptable, as linear regression is generally robust to moderate departures from normality. Homoscedasticity was assessed by plotting standardized residuals against fitted values, and no clear pattern suggestive of heteroscedasticity was observed. Multicollinearity among independent variables was evaluated using variance inflation factors (VIFs), with all VIF values below 2.0, indicating no substantial multicollinearity. Overall, the diagnostic results supported the appropriateness of using multivariable linear regression to examine associations between covariates and exercise-related KAP scores.

Several measures were implemented to minimize potential response bias, including social desirability bias. First, the questionnaire was administered anonymously using an online platform, and no personally identifiable information was collected. Participants were explicitly informed that their responses would be used solely for research purposes and would not affect their clinical care. Second, standardized instructions emphasized the importance of providing honest answers rather than socially desirable responses. Third, investigators were not involved in the participants’ clinical treatment, reducing potential pressure to provide favorable responses. Together, these procedures were designed to mitigate response bias and enhance the validity of self-reported data.

### 2.5 Statistical methods

Data analyses were performed using SPSS version 27.0 (IBM Corp., Armonk, NY, United States). Categorical variables were summarized as frequencies and percentages, while continuous variables were expressed as mean ± standard deviation (SD). Group comparisons were conducted using independent-samples *t* tests or one-way analysis of variance (ANOVA), as appropriate. Multivariable analyses were carried out using linear regression models to examine factors associated with exercise-related Knowledge-Attitude-Practice (KAP) scores. Regression coefficients (B), standardized coefficients (*β*), 95% confidence intervals (95% CI), and corresponding *p* values were reported. All statistical tests were two-sided, and a *p* value < 0.05 was considered statistically significant.

### 2.6 Variable definition and coding

All variables included in the statistical analyses were predefined and coded prior to data analysis. The dependent variable was the total exercise Knowledge-Attitude-Practice (KAP) score, treated as a continuous variable. Independent variables included sociodemographic characteristics (long-term residence [urban = 0, rural = 1], educational attainment [ordinal variable from elementary school or below to postgraduate level], and monthly income [ordinal categories]), disease-related factors (presence of diabetes-related complications [no = 0, yes = 1]), behavioral factors (regular exercise habits [no = 0, yes = 1]), exposure to exercise-related health education (participation in diabetes-specific exercise health education programmes [no = 0, yes = 1]), and social support indicators (encouragement from family or friends to exercise [no = 0, yes = 1]). Categorical variables with more than two levels were entered into the regression model as ordinal variables based on their inherent order. All variables were checked for missing values prior to analysis, and no imputation was required due to complete questionnaire responses.

Selection of independent variables for the multivariable linear regression model was guided by both theoretical considerations and empirical evidence. Variables were identified *a priori* based on the Knowledge-Attitude-Practice (KAP) framework and previous literature on exercise behavior among patients with type 2 diabetes mellitus, including sociodemographic characteristics, disease-related factors, exercise-related behaviors, and social support indicators. In addition, variables that showed statistical significance in univariate analyses (*p* < 0.05) were further considered for inclusion in the multivariable model to ensure parsimony while retaining clinically meaningful predictors. All selected variables were entered simultaneously into the final model using the enter method.

### 2.7 Regression model construction and assumption testing

Multivariable linear regression analysis was conducted to examine factors independently associated with exercise-related knowledge, attitude, and practice (KAP) scores. All selected variables were entered simultaneously into the model using the enter method. Prior to model fitting, key assumptions of linear regression were evaluated, including linearity, distribution of residuals, homoscedasticity, and multicollinearity. Linearity between independent variables and the dependent variable was assessed using scatterplots and partial regression plots, which indicated no substantial departures from linear relationships. The distribution of standardized residuals was examined using histograms and normal Q-Q plots. Although minor deviations from perfect normality were observed, particularly in the distribution tails, residuals were approximately symmetrically distributed with a mean close to zero. Given the relatively large sample size (*n* = 383), these deviations were considered acceptable, as linear regression is generally robust to moderate violations of normality assumptions (14). Homoscedasticity was assessed by visual inspection of residual-versus-fitted value plots, which showed no clear evidence of heteroscedasticity. Multicollinearity among independent variables was evaluated using variance inflation factors (VIFs), with all VIF values below 2.0, indicating no substantial multicollinearity. Model goodness-of-fit was evaluated using the coefficient of determination (*R*^2^) and the F statistic. Regression results are presented as unstandardized coefficients (B) with corresponding 95% confidence intervals (95% CI) and *p* values.

### 2.8 Statistical power

To ensure adequate statistical power for the multivariable linear regression analyses, an *a priori* power analysis was performed using G*Power 3.1 software. Based on established recommendations for regression models (15), a medium effect size (*f^2^* = 0.15), an alpha level *(α)*of 0.05, and 10 predictors in the final model were specified. The power analysis indicated that a total sample of at least 118 participants was required to achieve statistical power greater than 80% (1−*β* > 0.80) to detect moderate associations (16). The actual sample of 383 participants substantially exceeded this threshold, thus providing adequate power and supporting the stability and reproducibility of the regression results.

### 2.9 Ethical considerations

This study was approved by the Ethics Committee of the General Hospital of the Southern Theater Command of the People’s Liberation Army (Approval No. NZLLKZ2024155). Written informed consent was obtained from all participants prior to data collection.

## 3 Results

### 3.1 General characteristics of T2DM patients

This study included 383 subjects, comprising 249 males (65.0%) and 134 females (35.0%); other general characteristics are detailed in Table 1.

**Table 1:** Factors associated with KAP scores for physical activity among T2DM patients with different characteristics (*n* = 383).

Item	Category	Cases (%)	AP score (Mean ± SD)	Statistical value *(t/F)*	*p*-value
Gender				*t =* 0.437	0.663
	Male	249 (65.0%)	123.29 ± 24.22		
	Female	134 (35.0%)	122.15 ± 24.61		
Long-term residence				*t =* 2.819	0.005
	Urban	234 (61.1%)	125.66 ± 23.02		
	Rural	149 (38.9%)	118.54 ± 25.75		
Education attainment				*F =* 4.451	0.004
	Elementary school or below	97 (25.3%)	115.93 ± 24.72		
	Junior high and high school	134 (35.0%)	123.09 ± 22.94		
	University (Bachelor’s/Associate’s)	99 (25.8%)	126.27 ± 24.52		
	Graduate and above	53 (13.9%)	128.81 ± 24.38		
Monthly income				*F =* 3.759	0.005
	<3,000 yuan	78 (20.4%)	115.40 ± 24.90		
	3,000–5,000 yuan	114 (29.8%)	121.89 ± 24.03		
	5,000–8,000 yuan	111 (29.0%)	124.84 ± 24.18		
	8,000–20,000 yuan	48 (12.5%)	126.27 ± 22.09		
	>20,000 yuan	32 (8.4%)	132.88 ± 23.61		
Sedentary				*t =* 2.062	0.040
	Yes	196 (51.2%)	120.41 ± 26.55		
	No	187 (48.8%)	125.49 ± 21.54		
Body mass index (BMI)				*F =* 9.574	<0.001
	Underweight	14 (3.7%)	139.50 ± 29.98		
	Normal	312 (81.4%)	124.68 ± 22.62		
	Overweight	39 (10.2%)	110.08 ± 27.35		
	Obese	18 (4.7%)	106.78 ± 25.48		
Complications				*t =* 7.844	<0.001
	Yes	332 (86.7%)	126.66 ± 22.07		
	No	51 (13.3%)	98.33 ± 24.30		
Regular exercise habits				*t =* 5.724	<0.001
	Yes	146 (38.1%)	131.60 ± 21.33		
	No	237 (61.9%)	117.52 ± 24.56		
Participated in diabetes exercise health education				*t =* 8.290	<0.001
	Yes	339 (88.5%)	126.31 ± 21.97		
	No	44 (11.5%)	96.52 ± 25.77		
Family or friends encouraging exercise				*t =* 6.253	<0.001
	Yes	132 (34.5%)	133.11 ± 21.49		
	No	251 (65.5%)	117.51 ± 24.05		

### 3.2 Exercise KAP scores in T2DM patients

For the 383 T2DM participants, the overall exercise KAP score was 122.89 ± 24.33, corresponding to a score rate of 66.43% details are provided in Table 2. The top three and bottom three items across each dimension are presented in Table 3.

**Table 2:** Exercise-related knowledge, attitude, and practice (KAP) scores among patients with T2DM (*n* = 383).

Dimension	Possible score range	Mean ± SD	Score rate (%)
Total KAP score	37–185	122.89 ± 24.33	66.43
Exercise knowledge	15–75	49.10 ± 13.82	54.56
Exercise attitude	8–40	26.45 ± 7.66	66.1
Exercise practice	14–70	47.34 ± 12.55	67.63

Score rate (%) = (mean score/maximum possible score) × 100.

**Table 3:** Top and bottom scoring items of exercise-related KAP dimensions.

Dimension	Top-scoring items (Mean ± SD)	Bottom-scoring items (Mean ± SD)
Exercise knowledge	Awareness of hypoglycemia risk during or after exercise (3.52 ± 1.20)	Knowledge of resistance training prescription (2.98 ± 1.30)
	Awareness of heat stress and dehydration during exercise (3.48 ± 1.47)	Knowledge of balance exercise forms and frequency (3.00 ± 1.32)
	Understanding the role of exercise in glycaemic control (3.42 ± 1.21)	Knowledge of aerobic exercise prescription (3.02 ± 1.31)
Exercise attitude	Belief in the necessity of personalized exercise plans (3.46 ± 1.22)	Confidence in overcoming difficulties to maintain exercise (3.09 ± 1.18)
	Willingness to learn exercise knowledge through multiple channels (3.25 ± 1.19)	Belief that exercise is as important as medication (3.11 ± 1.24)
	Willingness to discuss and encourage exercise with others (3.20 ± 1.25)	Belief that household chores cannot replace exercise (3.13 ± 1.21)
Exercise practice	Seeking medical assistance for persistent discomfort (3.68 ± 1.05)	Maintaining diverse exercise routines (3.00 ± 1.25)
	Stopping exercise immediately when feeling unwell (3.65 ± 1.12)	Exercising 90 min after meals (3.05 ± 1.26)
	Carrying carbohydrates during exercise (3.49 ± 1.17)	Each exercise session lasting 30–60 min (3.10 ± 1.31)

Items were rated on a 5-point Likert scale (1 = strongly disagree to 5 = strongly agree).

### 3.3 Comparison of total exercise KAP scores among T2DM patients with different characteristics

Univariate analysis results indicated that the exercise-related KAP (Knowledge, Attitude, Practice) levels of patients with type 2 diabetes mellitus (T2DM) exhibited statistically significant differences across subgroups stratified by different demographic characteristics (*p* < 0.05). Specifically, the factors associated with these discrepancies included permanent residence, educational attainment, monthly income level, sedentary lifestyle, body mass index (BMI), presence of complications, regular exercise habits, participation in diabetes exercise health education programmes, and availability of exercise encouragement from family or friends. See Table 1 for details; other secondary variables are presented in Supplementary Table S1.

### 3.4 Multiple linear regression analysis

The total exercise Knowledge–Attitude–Practice (KAP) score was used as the dependent variable. Independent variables entered into the multivariable linear regression model included factors that were statistically significant in univariate analyses and considered theoretically relevant based on the Knowledge-Attitude-Practice framework. The overall regression model was statistically significant (*R*^2^ = 0.284, *F* = 16.476, *p* < 0.001), indicating that approximately 28.4% of the variance in exercise-related KAP scores among patients with type 2 diabetes mellitus (T2DM) was explained by the included predictors. After adjustment for covariates, higher educational attainment (B = 2.34, 95% CI: 0.16 to 4.52), higher monthly income (B = 2.51, 95% CI: 0.70 to 4.33), presence of diabetes-related complications (B = 13.01, 95% CI: 3.95 to 22.06), having regular exercise habits (B = 6.45, 95% CI: 1.78 to 11.11), participation in diabetes-specific exercise health education programmes (B = 14.00, 95% CI: 4.45 to 23.55), and encouragement from family or friends to exercise (B = 7.24, 95% CI: 2.48 to 12.00) were independently associated with higher exercise KAP scores (all *p* < 0.05). In contrast, long-term rural residence was negatively associated with exercise-related KAP levels (B = −4.86, 95% CI: −9.27 to −0.44, *p* = 0.031). No evidence of multicollinearity was observed among independent variables. Detailed regression coefficients, standardized coefficients, confidence intervals, and significance levels are presented in Table 4.

**Table 4:** Results of linear regression analysis on factors associated with KAP regarding exercise in patients with type 2 diabetes mellitus.

Independent variable	*B* value	SE	*β* value	*t*-value	*P*-value	95%CI	
						Lower bound	Upper bound
Constant	82.041	9.698		8.459	<0.001		
long-term residence	−4.856	2.244	−0.097	−2.164	0.031	−9.27	−0.44
Educational attainment	2.339	1.107	0.096	2.113	0.035	0.16	4.52
Monthly income	2.514	0.921	0.122	2.730	0.007	0.70	4.33
Complications	13.005	4.606	0.182	2.824	0.005	3.95	22.06
Regular exercise habits	6.445	2.372	0.129	2.717	0.007	1.78	11.11
Participated in diabetes exercise health education	13.996	4.857	0.184	2.881	0.004	4.45	23.55
Family or friends encouraging exercise	7.240	2.422	0.142	2.989	0.003	2.48	12.00

## 4 Discussion

### 4.1 Low overall levels of KAP regarding exercise among T2DM patients indicate significant demand for exercise health education

In this study, the overall exercise-related Knowledge-Attitude-Practice (KAP) score among hospitalized patients with type 2 diabetes mellitus (T2DM) was moderate but suboptimal, with the knowledge dimension scoring notably lower than attitude and practice. This imbalance suggests that although patients may recognize the general importance of physical activity, insufficient and fragmented exercise knowledge remains a key barrier to the initiation and maintenance of appropriate exercise behaviors. Specifically, patients demonstrated basic awareness of exercise-related risks and the role of physical activity in glycaemic control, yet lacked adequate understanding of core components of exercise prescription, including modality, intensity, frequency, and duration. Such deficits are clinically relevant, as inadequate knowledge of exercise structure may undermine patients’ confidence and ability to engage in sustained physical activity, a finding consistent with previous qualitative and quantitative research (17). Attitudinal responses revealed a similarly complex pattern. While participants generally endorsed the necessity of personalized exercise plans and expressed willingness to seek exercise-related information, misconceptions persisted regarding the substitutability of daily chores for structured exercise and the relative importance of exercise compared with pharmacological treatment. These inconsistent beliefs indicate an unstable exercise attitude, which may translate into irregular or insufficient exercise engagement. Prior studies have reported that disease-related stigma, medication dependence, and misperceptions about physical activity contribute to ambivalent or negative exercise attitudes among individuals with T2DM (18). In terms of practice, patients reported relatively high adherence to safety-related behaviors, such as monitoring discomfort and carrying carbohydrates during exercise, but scored lower on items reflecting exercise planning, timing, and diversity. This pattern suggests that patients tend to prioritize immediate risk avoidance over long-term exercise optimization. Such behavior may be partly attributable to reliance on non-systematic information sources, including unfiltered online content, which can lead to inconsistent or inaccurate exercise practices (16, 17). Taken together, these findings indicate that the primary challenge in exercise management for T2DM patients lies not merely in motivation, but in the lack of structured, evidence-based exercise knowledge that links awareness to effective behavior. Consistent with the Chinese Guidelines for Exercise Therapy in Type 2 Diabetes (2024 Edition) (19), targeted exercise education should emphasize practical exercise prescription principles and individualized guidance, rather than general encouragement alone. Addressing these knowledge gaps may help stabilize exercise attitudes and promote more consistent and effective exercise practices.

### 4.2 Personal characteristics correlated with KAP regarding physical activity among T2DM patients: long-term residence, educational attainment, monthly income, and comorbidities

This study identified long-term place of residence, educational attainment, monthly income, and the presence of diabetes-related complications as independent correlates of exercise-related KAP levels among hospitalized patients with T2DM. These factors reflect broader structural, cognitive, and clinical influences on exercise-related health behaviors rather than isolated individual choices. Patients residing in urban areas demonstrated higher exercise-related KAP scores than those living in rural settings. This finding is consistent with previous evidence indicating that urban environments provide greater access to healthcare resources, structured health education, and physical activity infrastructure, thereby facilitating both exposure to exercise-related knowledge and opportunities for implementation (19, 20). In contrast, rural populations often face limited access to professional exercise guidance and fewer supportive environments for physical activity, reinforcing persistent urban–rural disparities in health behaviors (11). Notably, the magnitude of the observed association suggests that residence functions as a contextual determinant of exercise behavior, underscoring the need for place-sensitive intervention strategies rather than uniform health education approaches. Educational attainment was positively associated with exercise-related KAP, indicating that cognitive and informational capacity plays a critical role in translating general health messages into actionable exercise behaviors. Individuals with higher educational levels may be better equipped to access, interpret, and critically evaluate health information from professional or authoritative sources (21), whereas those with lower educational backgrounds may encounter greater difficulty distinguishing evidence-based guidance from misleading or oversimplified information (22). Importantly, the relatively modest effect size observed in this study suggests that education alone is insufficient to ensure optimal exercise behavior, highlighting the importance of tailoring exercise education materials to different levels of health literacy. Monthly income also showed an independent association with KAP scores. Higher-income patients may perceive exercise as a long-term investment in disease management and prevention, thereby demonstrating stronger engagement with exercise-related behaviors (23). Conversely, financially constrained individuals may limit physical activity due to concerns about potential costs, injury-related expenses, or competing socioeconomic priorities, which may attenuate the effectiveness of exercise recommendations (24). These findings support the prioritization of low-cost, accessible exercise interventions for economically disadvantaged populations. Interestingly, patients with diabetes-related complications exhibited higher exercise-related KAP levels than those without complications. While complications may impose physical limitations that restrict exercise capacity (25). they may simultaneously increase risk awareness and perceived disease severity, motivating greater engagement with exercise-related knowledge and health behaviors (26). This paradox highlights the complexity of behavior change in chronic disease management and suggests that clinical deterioration may act as a catalyst for health behavior engagement rather than a barrier alone.

Overall, these findings indicate that exercise-related KAP among T2DM patients is shaped by a combination of socioeconomic context, cognitive resources, and clinical experience. Interventions aimed at improving exercise behavior should therefore move beyond generic education models and incorporate stratified strategies that address structural inequities, health literacy, and disease perception.

### 4.3 Exercise habits, health education, and family support are crucial factors associated with the KAP levels of physical activity among T2DM patients

Regular exercise habits, participation in structured exercise health education, and encouragement from family or friends emerged as key modifiable factors associated with exercise-related KAP levels among patients with T2DM. These factors operate through experiential learning, cognitive reinforcement, and social facilitation, collectively shaping patients’ engagement with physical activity. Patients who maintain regular exercise routines are more likely to experience tangible benefits such as improved physical fitness and glycaemic stability, which can reinforce positive beliefs regarding exercise efficacy and strengthen adherence over time (18). Through repeated practice, patients also acquire practical skills such as adjusting exercise intensity, selecting appropriate timing, and recognizing warning signs thereby transforming abstract knowledge into actionable competence (26). In contrast, patients lacking sustained exercise experience may remain uncertain about exercise benefits, perpetuating a cycle of limited knowledge, unstable attitudes, and insufficient practice. These findings suggest that initial engagement in exercise, even at low intensity, may play a critical role in activating positive feedback loops that support long-term behavior change. Participation in diabetes-specific exercise health education programmes was strongly associated with higher KAP levels. Such programmes, typically delivered by trained healthcare professionals, provide standardized and evidence-based guidance that can correct misconceptions and reduce uncertainty surrounding exercise prescription (14, 25). Importantly, the observed association underscores that effective education extends beyond generic encouragement and requires personalized recommendations tailored to patients’ clinical status and capabilities. This highlights the role of healthcare professionals not only as information providers but also as facilitators of behavioral self-efficacy.

Social support, particularly encouragement from family members or peers, was also independently associated with improved exercise-related KAP. Supportive social environments may enhance motivation, accountability, and emotional reassurance, thereby lowering psychological and practical barriers to exercise participation (27). Family involvement in diabetes management has been shown to promote adherence to lifestyle interventions and foster more sustainable behavior change (28). Integrating family-oriented strategies into exercise interventions may therefore amplify their effectiveness by aligning individual motivation with social reinforcement. Taken together, these findings indicate that improving exercise-related KAP among T2DM patients requires coordinated interventions that combine habit formation, structured professional education, and social support. However, these associations should be interpreted cautiously. Although the sample size provided adequate statistical power to detect moderate effects, the cross-sectional design precludes causal inference, and unmeasured psychosocial or environmental factors may account for part of the observed variability. Furthermore, as the study population consisted exclusively of hospitalized patients, these findings may not be fully generalizable to community-dwelling or outpatient T2DM populations. These limitations are addressed in the following section (29, 30).

### 4.4 Study limitations and implications for generalizability

Several limitations of this study should be acknowledged. First, the study population consisted exclusively of hospitalized patients with type 2 diabetes mellitus, which may introduce selection bias. Hospitalized individuals often have more severe disease, a higher burden of complications, and greater exposure to medical supervision and health education than community-dwelling patients. As a result, exercise-related knowledge, attitudes, and practices observed in this study may not fully reflect those of patients managed in outpatient or primary care settings. Second, the cross-sectional design limits causal inference. Although multiple sociodemographic, clinical, and behavioral factors were identified as being associated with exercise-related KAP levels, the temporal direction of these relationships cannot be determined. It is therefore possible that higher KAP levels both influence and are influenced by exercise engagement and health education exposure. Third, although the sample size provided adequate statistical power to detect moderate associations, the absence of significant associations for certain variables does not exclude the possibility of type II error, particularly for factors with small effect sizes or limited variability. In addition, the regression model explained approximately 28.4% of the variance in KAP scores, indicating that unmeasured factors such as psychological readiness, environmental constraints, or cultural influences may also play an important role in shaping exercise behavior. Finally, data were collected using a self-reported questionnaire, which may be subject to reporting and social desirability bias despite measures taken to ensure anonymity and encourage honest responses. Future studies employing longitudinal designs, objective measures of physical activity, and broader recruitment from community and outpatient populations are warranted to validate and extend these findings.

## 5 Conclusion

In summary, this study demonstrates that exercise-related knowledge, attitudes, and practices among hospitalized patients with type 2 diabetes mellitus remain suboptimal, with knowledge deficits representing a particularly prominent gap. Exercise-related KAP levels were associated with a combination of sociodemographic, clinical, and behavioral factors, including place of residence, educational attainment, monthly income, presence of diabetes-related complications, regular exercise habits, participation in diabetes-specific exercise education, and encouragement from family or peers. These findings highlight that improving exercise behavior in patients with T2DM requires more than general recommendations. Structured, evidence-based exercise education, support for habit formation, and the integration of family and social support appear to be important components of effective exercise management strategies. Interventions tailored to patients’ socioeconomic context and health literacy may help bridge the gap between awareness and sustained practice. While causal inferences cannot be drawn, the present study provides empirical evidence to inform the design of targeted exercise education and support programmes for T2DM patients in clinical settings. Future longitudinal and intervention studies involving broader patient populations are warranted to further clarify causal pathways and evaluate the effectiveness of tailored exercise-based interventions.

## Data Availability

The raw data supporting the conclusions of this article will be made available by the authors, without undue reservation.
