# Supplementary material for: Analysis of knowledge, attitudes and practices regarding exercise among type 2 diabetes patients and influencing factors: a cross-sectional study
Source: Front Med (Lausanne). 2026 Feb 10;13:1766153. doi: 10.3389/fmed.2026.1766153 (PMC12929124; doi:10.3389/fmed.2026.1766153)
Supplement: Supplementary file 1 [file Table_1.docx]

Table S1. Factors associated with KAP Scores for Physical Activity Among T2DM Patients with Different Characteristics (n=383)

| Item |  | Cases (%) | Item Mean Score (*‾ x±s*) | Statistical Value *(F/t)* | P-value |
| --- | --- | --- | --- | --- | --- |
| Age |  |  |  | *F =* 1.400 | 0.248 |
|  | 18-30 | 3 (0.7%) | 109.33±4.93 |  |  |
|  | 31-50 | 277 (72.3%) | 121.96±25.02 |  |  |
|  | ≥51 | 103 (27.0%) | 125.79±22.51 |  |  |
| Housing Status |  |  |  | *t =* -0.743 | 0.458 |
|  | Living Alone | 163 (42.3%) | 121.82±24.29 |  |  |
|  | Non-single living | 220 (57.7%) | 123.69±24.39 |  |  |
| Marital Status |  |  |  | *F =* 0.205 | 0.815 |
|  | Married | 263 (68.7%) | 122.51 ± 24.79 |  |  |
|  | Unmarried | 114 (29.8%) | 123.96 ± 23.86 |  |  |
|  | Other | 6 (1.5%) | 119.33±11.11 |  |  |
| Occupation |  |  |  | *F =* 1.629 | 0.115 |
|  | Enterprises/Public Institutions/Civil Servants | 76 (19.8%) | 117.50±26.90 |  |  |
|  | Military Personnel | 24 (6.3%) | 122.33±22.62 |  |  |
|  | Self-employed | 66 (17.2%) | 125.71±24.86 |  |  |
|  | Healthcare professionals | 56 (14.6%) | 118.79±23.90 |  |  |
|  | Retired | 27 (7.0%) | 129.04 ± 19.36 |  |  |
|  | Migrant workers | 93 (24.3%) | 123.57±24.69 |  |  |
|  | Agricultural labor | 21 (5.5%) | 133.43±19.04 |  |  |
|  | Unemployed | 20 (5.2%) | 122.53±20.93 |  |  |
|  | >20,000 yuan | 32 (8.4%) | 132.88 ± 23.61 |  |  |
| Duration of Illness |  |  |  | *F = 2.522* | 0.058 |
|  | ＜1 year | 103 (26.9%) | 121.85±24.03 |  |  |
|  | 1–5 years | 134 (35.0%) | 125.59±23.47 |  |  |
|  | 6–10 years | 99 (25.8%) | 124.21±25.38 |  |  |
|  | >10 years | 47 (12.3%) | 114.68±23.99 |  |  |
| Number of hospitalizations |  |  |  | *F =* 0.538 | 0.584 |
|  | 1–3 times | 330 (86.2%) | 122.58 ± 24.76 |  |  |
|  | 4-5 times | 49 (12.85) | 124.06±21.98 |  |  |
|  | >5 times | 4 (1.0%) | 134.50±14.46 |  |  |
| Family history |  |  |  | *t =* -0.814 | 0.416 |
|  | Yes | 137 (35.8%) | 121.53±26.20 |  |  |
|  | No | 246 (64.2%) | 123.65±23.25 |  |  |
| Has diabetes complications appeared in family members or friends? |  |  |  | *t =* 0.546 | 0.586 |
|  | Yes | 186 (48.6%) | 123.59 ± 25.66 |  |  |
|  | No | 197 (51.4%) | 122.23±23.05 |  |  |
| History of Sports Injury |  |  |  | *t =* -0.564 | 0.573 |
|  | Yes | 166 (43.3%) | 122.07 ± 26.22 |  |  |
|  | No | 217 (56.7%) | 123.52±22.82 |  |  |
| Is there professional management or follow-up of exercise? |  |  |  | *t =* -0.710 | 0.478 |
|  | Yes | 195 (51.0) | 122.03±26.00 |  |  |
|  | No | 188 (49.0) | 123.78±22.50 |  |  |
| Has a personalized exercise plan been developed by a professional? |  |  |  | *t =* -0.393 | 0.694 |
|  | Yes | 148 (38.6%) | 122.25 ± 26.79 |  |  |
|  | No | 235 (61.4%) | 123.29±22.70 |  |  |
